# Prediction of bronchopulmonary dysplasia in very preterm infants: competitive risk model nomogram

**DOI:** 10.3389/fped.2024.1335891

**Published:** 2024-02-20

**Authors:** Andrea Sucasas-Alonso, Sonia Pértega-Díaz, Vanesa Balboa-Barreiro, Fermín García-Muñoz Rodrigo, Alejandro Avila-Alvarez

**Affiliations:** ^1^ Neonatology Department, Complexo Hospitalario Universitario de A Coruña, A Coruña, Spain; ^2^ Rheumatology and Health Research Group, Department of Health Sciences, Universidade da Coruña, Ferrol, Spain; ^3^ Nursing and Health Care Research Group, Instituto de Investigación Biomédica de A Coruña (INIBIC), A Coruña, Spain; ^4^ Research Support Unit, Complexo Hospitalario Universitario A Coruña, A Coruña, Spain; ^5^ Division of Neonatology, Complejo Hospitalario Universitario Insular Materno-Infantil, Las Palmas de Gran Canaria, Las Palmas, Spain

**Keywords:** bronchopulmonary dysplasia, prediction, death, mechanical ventilation, preterm

## Abstract

**Objective:**

To develop predictive clinical models of bronchopulmonary dysplasia (BPD) through competing risk analysis.

**Methods:**

Retrospective observational cohort study, including preterm newborns ≤32 weeks gestational age, conducted between January 1, 2013 and September 30, 2022 in a third-level Neonatal Intensive Care Unit in Spain. A prediction study was carried out using competing risk models, where the event of interest was BPD and the competing event was death. A multivariate competing risk model was developed separately for each postnatal day (days 1, 3, 7 and 14). Nomograms to predict BPD risk were developed from the coefficients of the final models and internally validated.

**Results:**

A total of 306 patients were included in the study, of which 73 (23.9%) developed BPD and 29 (9.5%) died. On day 1, the model with the greatest predictive capacity was that including birth weight, days since rupture of membranes, and surfactant requirement (area under the receiver operating characteristic (ROC) curve (AUC), 0.896; 95% CI, 0.792–0.999). On day 3, the final predictive model was based on the variables birth weight, surfactant requirement, and Fraction of Inspired Oxygen (FiO_2_) (AUC, 0.891; 95% CI, 0.792–0.989).

**Conclusions:**

Competing risk analysis allowed accurate prediction of BPD, avoiding the potential bias resulting from the exclusion of deceased newborns or the use of combined outcomes. The resulting models are based on clinical variables measured at bedside during the first 3 days of life, can be easily implemented in clinical practice, and can enable earlier identification of patients at high risk of BPD.

## 1 Introduction

Bronchopulmonary dysplasia (BPD) is a common chronic lung disease in premature infants that leads to significant morbidity and mortality (1–3). Although multiple studies have sought to develop effective preventive interventions for BPD, including postnatal corticosteroids and avoidance of invasive mechanical ventilation (4), the incidence of BDP does not appear to have declined in recent years (5, 6).

Inflammation and oxidative stress that ultimately lead to BPD appear to occur early in the neonatal period, preceding clinical symptoms of BPD and diagnosis based on current definitions (7). Moreover, the description of different BPD phenotypes highlights the need to individualize care and, potentially, to develop more effective preventive strategies (8). Therefore, early and accurate identification of babies who will later develop BPD is among the highest priorities in modern neonatal care. Good-quality predictive models for BPD aim to establish an individual risk of BPD, helping clinicians to identify high-risk patients and individualize care, and hopefully enabling more effective preventive strategies. While a plethora of predictive models have been developed in recent decades (9), none are fully incorporated into routine clinical practice (5, 10) and all suffer from a high risk of bias (11).

Heterogeneity in clinical practices across units and countries, the unavailability of certain variables used in models, the complexity of some models, their development based on population data from decades ago, and a lack of external validation are just some of the factors that may have contributed to the apparent failure in developing an optimal prediction tool. Furthermore, certain methodological issues need to be critically evaluated. Most models were developed using logistic regression analysis of datasets that either exclude infants who died before BPD diagnosis, or use a combined outcome of mortality and BPD (12–16).

This approach attributes equal weight to outcomes that are clearly different for both clinicians and parents (death and BPD) and, more problematically, analyzes predictors of BPD in a lower-risk infant population, eliminating from the analysis those children who die before 36 weeks of postmenstrual age (PMA). Although BPD is usually defined at a fixed point in time (typically 36 weeks PMA), it could be considered a time-to-event outcome, diagnosed at some point after birth, meaning that it could be correctly studied using survival analysis methods. Competing risks analysis, a special type of survival analysis, is particularly useful when patients are at risk of more than one mutually exclusive event, such as BPD and death (17).

In the present study we sought to develop an up-to-date model for early prediction of BPD, based on routinely collected variables, and to explore the utility of competing risk survival analysis methods in this context. We hypothesized that this methodology may constitute a superior means of studying the risk of BPD, overcoming the methodological limitations of other approaches.

## 2 Materials and methods

This single-center retrospective analysis of data recorded in a prospective registry was conducted between January 1, 2013 and September 30, 2022, at a third-level Neonatal Intensive Care Unit of a hospital of the Spanish Public Health System. Preterm infants ≤32 weeks gestational age (GA) were included in the study. Newborns with major congenital anomalies were excluded.

Variables related to prenatal and obstetric characteristics, demographics, resuscitation in the delivery room, respiratory support, and neonatal evolution during first hospital admission were collected. The main prematurity-related outcomes were recorded.

Gestational age was estimated based on the last menstrual period date and/or obstetric and ultrasound parameters recorded in the maternal medical record. BPD was defined as the need for supplemental oxygen therapy for 28 days of life and was classified based on oxygen requirements and respiratory support at 36 weeks of PMA (18). Patent ductus arteriosus was diagnosed by cardiac ultrasound and managed according to local protocols based on echocardiographic and clinical data (only ductus >1.5 mm in patients on respiratory support were considered) (19). Maternal chorioamnionitis was defined by clinical diagnosis in the medical record or by histologic diagnosis by placental pathology (20–22). Nosocomial infection was defined as positive microbial growth on one or more bloodstream cultures or any sterile body fluid obtained after 72 h of life with accompanying clinical signs of sepsis (23). NEC grade ≥2 according to the Bell classification was considered (24, 25). Intraventricular hemorrhage (IVH) was defined and graded according to Volpe (26, 27). All newborns were screened for retinopathy of prematurity and its grade was classified according to international guidelines (28). Intrauterine growth restriction was described as birth weight ≤1.5 *z*-score according to Fenton growth charts (29).

There were standard guidelines for respiratory management in place in the NICU during the study period, which were in keeping with European and national recommendations (30, 31). In brief, stabilization in the delivery room as initiated with mask and T-resuscitator applying CPAP or NIPPV depending on the presence of spontaneous breathing and heart rate. NIPPV was used in the NICU as early rescue therapy before considering orotracheal intubation or as initial support at clinical discretion. Surfactant was administered using the INSURE (intubation-surfactant-extubation) procedure in the first hours of life when FiO_2_ requirements were greater than 30% on NIV or if the infant required orotracheal intubation for any other reason. For the INSURE technique, premedication with caffeine, fentanyl and atropine was administered. Caffeine citrate was given prophylactically to all infants <28 weeks GA and in those <32 weeks who developed apneas (20 mg/Kg bolus followed by 5 mg/Kg per day, which can be increased to 10 mg/Kg if clinically needed). Hydrocortisone administration was considered in infants on IMV and FiO2 >0.3 after the first week of life. According with the European guidelines, the oxygen saturation target was established between 90% and 94%.

No relevant changes were made in clinical practice during the study period.

The study was approved by the local Research Ethics Committee (code 2017/360).

### 2.1 Statistical analysis

Maternal and perinatal characteristics were described and compared between neonates without BPD, neonates with BPD, and neonates who died. A BPD prediction study was carried out using competing risk analysis, where the event of interest was BPD and the competing event was death (17). PMA was considered as the time variable, and each newborn was followed until death or hospital discharge.

The cumulative incidence function (CIF) of both BPD and death was estimated, using the method proposed by Kalbfleish and Prentice (32). Fine-Gray univariate and multivariate competing risk models were used to identify predictive factors for BPD and death separately (17). Variables considered clinically important and those for which a statistically significant difference (*p* < 0.05) was observed in the univariate analysis were included in the multivariate models, using Akaike information criterion (AIC) as the selection criterion in a stepwise forward selection strategy. A multivariate competing risk model was developed separately for each timepoint (days 1, 3, 7 and 14) using data from infants who survived to the prediction day. For each day, the final prediction model was the simplest model that included variables significantly associated with BPD among those with the greatest predictive ability.

Nomograms for predicting BPD risk were developed from the coefficients of the final models and were internally validated. The primary event was BPD (defined as the need for supplemental oxygen therapy for 28 days of life), and the estimated cumulative probability of BPD at 36 weeks was considered the prediction of interest, since all the babies who develop BPD do so before this time horizon. The time-dependent area under the receiver operating characteristic (ROC) curve (AUC) for *t* = 36 weeks was used to quantify the discrimination performance of the competing risk nomograms (33), since the concordance index is inadequate when the aim is to predict the risk of an event at a fixed timepoint. A 10-fold cross-validation was used to obtain an optimism-adjusted AUC.

To further analyze the models' discriminative ability, newborns were divided into four risk groups based on their estimated probability of BPD according to both nomograms. Gray's test was used to compare the cumulative incidence of BPD between groups (34). Bootstrap cross-validation calibration plots (*B* = 100 resamples) were also used to measure the degree of consistency between predicted and observed BPD probabilities. Finally, the clinical utility and overall advantages of the nomograms were assessed using decision curve analysis (DCA) (35).

All analyses were performed using the statistical software SPSS v.28 and R v4.2. The “*cmprsk*”*,* “*riskRegression*”*,* and “*pec*” packages were used for competing risk analysis and internal validation of the models. Nomograms were built with the “*QHScrnomo*” package. DCA was implemented using the “*dcurves*” package.

All reported significance levels are two-sided. Statistical significance was defined as a *p*-value < 0.05.

## 3 Results

**Figure 1:**
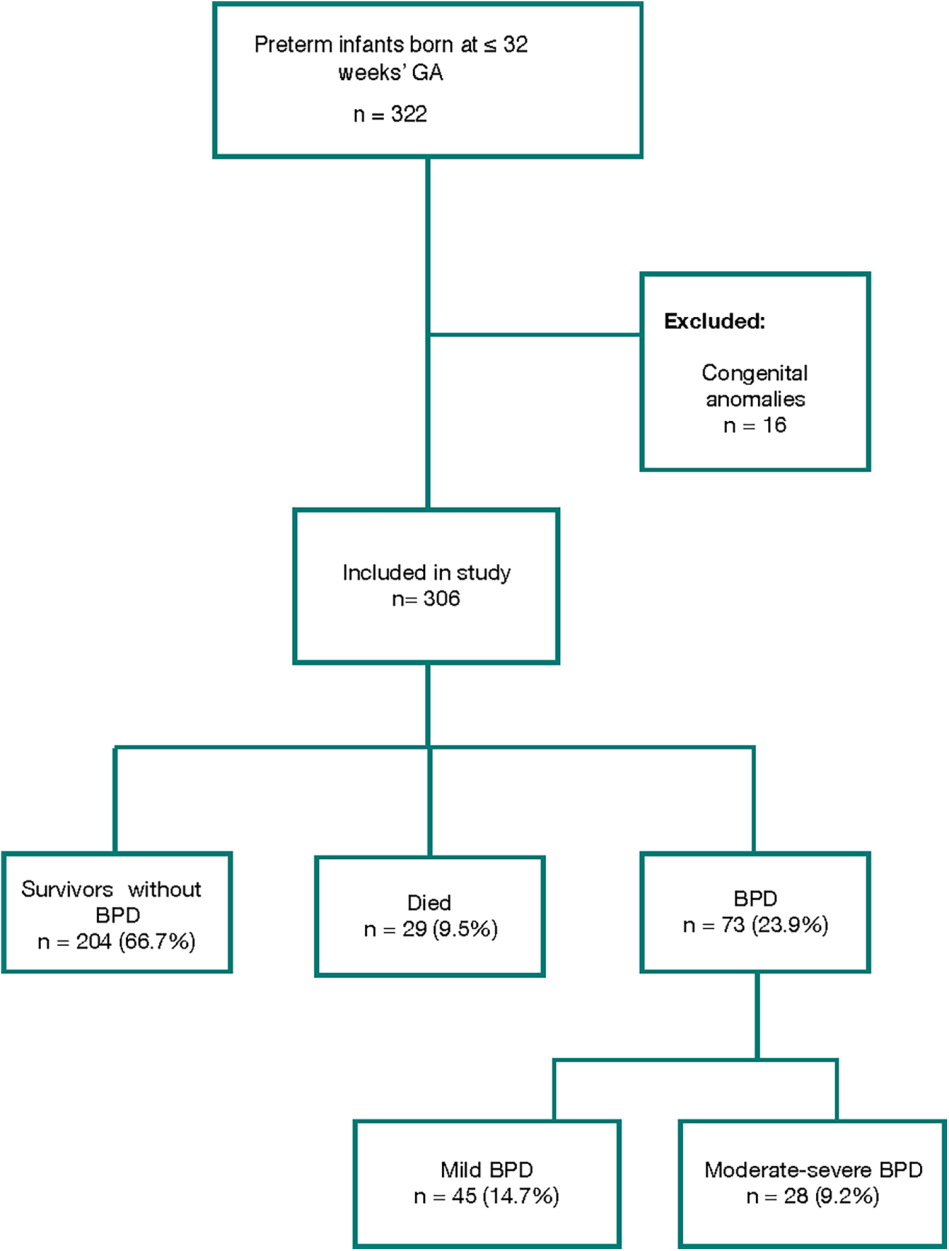
Flowchart of study population selection and diagnosis of bronchopulmonary dysplasia (BPD).

During the study period, 322 preterm newborns ≤32 weeks GA born at the study hospital were evaluated. Sixteen patients with major congenital anomalies were excluded. Ultimately, 306 patients were included in the study, of whom 73 (23.9%) developed BPD and 29 died (9.5%) (Figure 1, Supplementary Figure S1).

Mean GA and birth weight were 29.3 ± 2.3 weeks and 1,135.8 ± 309.7 g, respectively. Table 1 shows the most relevant characteristics of the study population. Results of the univariate analysis of factors associated with either BPD or death are shown in Table 2 and Supplementary Table S1.

**Table 1:** Maternal and neonatal characteristics of the study cohort.

Gestational age (weeks)	29.3 ± 2.3 (27.6–31.1)
Gestational age <28 weeks	90 (29.4)
Maternal age (years)	36.6 ± 42.7 (30–39)
Maternal arterial hypertension	69 (22.5)
Multiple birth	112 (36.6)
IVF	62 (20.3)
Female	158 (51.6)
Prenatal steroids	219 (95.1)
Intrauterine growth restriction	56 (18.3)
Chorioamnionitis	51 (16.7)
Caesarean section	224 (73.2)
Birth weight (grams)	1,135.8 ± 309.7 (910–1,400)
Birth weight *z*-score	−0.9 ± 7.8 (−1 to 0.11)
Surfactant	159 (52)
NIV during admission	283 (92.5)
MV during admission	124 (40.5)
Duration of MV (hours)	165.5 ± 241.8 (20–216)
Duration of NIV (hours)	182.5 ± 193.4 (48–235)
Duration of supplementary oxygen (hours)	564.3 ± 689.84 (57.7–1,008)
BPD	73 (23.9)
Moderate-severe BPD	28 (9.2)
Postnatal steroids	39 (12.7)
Late-onset sepsis	85 (27.8)
Medically treated PDA	38 (12.4)
Surgically treated PDA	17 (5.6)
IVH >grade II	20 (6.5)
Surgical NEC	11 (3.6)
Leukomalacia	19 (6.2)
ROP grade >II	16 (5.2)

Values are expressed as *n* (%) for qualitative variables and mean ± standard deviation (interquartile range) for quantitative variables. IVF, *in vitro* fertilization; MV, mechanical ventilation; NIV, non-invasive mechanical ventilation; IVH, intraventricular hemorrhage; NEC, necrotizing enterocolitis; BPD, bronchopulmonary dysplasia; PDA, patent ductus arteriosus; ROP, retinopathy of prematurity.

**Table 2:** Univariate competing-risk regression to identify risk factors for bronchopulmonary dysplasia (BPD).

	Survival without BPD	Survival with BPD	Death	BPD-sHR (95% CI)	*p*-value
Day of life 1					
Gestational age (weeks)	27.4 ± 1.7	30.4 ± 1.6	26.6 ± 1.8	–	–
Maternal age (years)	34.1 ± 5.9	43.6 ± 86.2	35.6 ± 5.8	0.97 (0.94–1.01)	0.250
Maternal arterial hypertension	42 (21.1)	19 (26)	8 (27.6)	1.17 (0.71–1.94)	0.520
Maternal smoking	27 (13.6)	7 (9.9)	2 (6.9)	0.76 (0.35–1.64)	0.500
Multiple birth	79 (39.7)	25 (34.2)	6 (20.7)	0.82 (0.51–1.32)	0.430
IVF	43 (21.6)	12 (16.4)	6 (20.7)	0.77 (0.41–1.44)	0.430
Female	107 (53.8)	34 (46.6)	13 (44.8)	0.79 (0.50–1.25)	0.320
Prenatal steroids	193 (97)	69 (94.5)	24 (82.8)	0.88 (0.33–2.35)	0.810
Chorioamnionitis	29 (14.6)	13 (17.8)	9 (31)	1.23 (0.65–2.32)	0.510
Caesarean section	151 (75.9)	48 (65.8)	22 (75.9)	0.57 (0.34–0.94)	0.030
Days since rupture of membrane	2.61 ± 9.23	4.14 ± 15.69	1.41 ± 4.15	1.01 (0.99–1.02)	0.067
Birth weight (grams)	1,271.7 ± 263.5	923.1 ± 207.9	775.5 ± 201.2	0.99 (0.99–0.99)	<0.001
Birth weight *z*-score	−1.21 ± 9.60	−0.28 ± 0.85	−0.63 ± 0.88	1.43 (1.08–1.89)	0.012
Apgar 1 min	6.79 ± 1.58	5.96 ± 1.92	4.86 ± 3.36	0.85 (0.76–0.96)	0.008
Apgar 5 min	8.15 ± 1.26	7.51 ± 1.62	6.66 ± 2.62	0.87 (0.79–0.97)	0.016
Oxygen in DR	160 (80.4)	69 (94.5)	27 (93.1)	3.22 (1.19–8.72)	0.021
Intubation in DR	25 (12.6)	25 (34.2)	11 (379)	2.33 (1.43–3.79)	0.001
Chest compressions in DR	9 (4.5)	4 (5.5)	6 (20.7)	0.87 (0.32–2.39)	0.800
Adrenaline in DR	7 (3.5)	4 (5.5)	4 (13.8)	1.19 (0.42–3.34)	0.730
Temperature on admission (°C)	35.94 ± 0.66	35.09 ± 4.11	35.16 ± 1.07	0.94 (0.92–0.96)	<0.001
Surfactant	65 (32.7)	60 (82.2)	27 (93.1)	5.13 (2.86–9.19)	<0.001
Age at surfactant administration (hours)	5.09 ± 9.38	2.03 ± 3.05	1.78 ± 2.63	0.92 (0.86–0.98)	0.014
Day of life 3					
FiO_2_	22.3 ± 3.2	27.1 ± 11.2	27.4 ± 6.0	1.03 (1.02–1.04)	<0.001
MV	5 (2.5)	21 (28.8)	13 (76.5)	3.16 (1.85–5.39)	<0.001
MV during the first 72 h of life	1 (0.5)	18 (24.7)	11 (57.9)	3.65 (2.05–6.48)	<0.001
PDA	3 (1.5)	6 (8.2)	1 (3.4)	3.38 (1.40–8.13)	0.006
Nosocomial infection	2 (1)	0	0	–	–
Day of life 7					
FiO_2_	21.5 ± 2.9	26.2 ± 7.1	37.6 ± 23.3	1.02 (1.00–1.05)	0.013
MV	2 (1)	14 (19.2)	8 (72.7)	3.98 (2.10–7.53)	<0.001
MV during the first week	0	7 (9.6)	5 (35.7)	3.34 (1.48–7.53)	0.004
PDA	14 (7)	12 (16.4)	4 (13.8)	1.84 (0.93–3.64)	0.078
Nosocomial infection	8 (4)	7 (9.6)	3 (10.3)	1.47 (0.70–3.07)	0.300
Day of life 14					
FiO_2_	21.27 ± 2.31	28.73 ± 10.02	45.33 ± 28.15	1.03 (1.01–1.06)	0.003
MV	1 (0.5)	18 (24.7)	4 (66.7)	7.03 (4.00–12.3)	0.000
MV during the first 2 weeks	0	5 (6.8)	2 (18.2)	4.72 (1.77–12.5)	0.002
MV during 3–14 days of life	0	6 (8.2)	3 (25)	4,17 (1,73–10,0)	0,001
PDA	16 (8.1)	21 (18.8)	5 (17.2)	2.79 (1.62–4.78)	0.000
Nosocomial infection	30 (15.1)	24 (32.9)	9 (31)	2.00 (1.23–3.27)	0.005

Values are expressed as *n* (%) for qualitative variables and mean ± SD (IQR) for quantitative variables. BPD-sHR, bronchopulmonary dysplasia-related subhazard ratio; CI, confidence interval; FiO2, fraction of inspired oxygen; IVF, *in vitro* fertilization; DR, delivery room; MV, mechanical ventilation; NIV, non-invasive mechanical ventilation; PDA, patent ductus arteriosus.

Table 3 shows the final models for BPD prediction. On day 1 the greatest predictive capacity was obtained using the variables birth weight, days since rupture of membranes, and surfactant requirement. On day 3, the final predictive model was established using the variables birth weight, surfactant requirement, and FiO_2._ For days 7 and 14, only birth weight and surfactant requirement were statistically significant in the multivariate analysis. In particular, FiO_2_ on day of life (DOL) 7 and 14 did not achieve statistical significance.

**Table 3:** Multivariate competing risk analysis of bronchopulmonary dysplasia (BPD) for different postnatal days of life.

	sHR (95% CI)	*P* value	AUC (95% CI)	
			Apparent	BCV-corrected
Day of life 1			0.896 (0.792–0.999)	0.881 (0.767–0.995)
Birth weight (grams)	0.99 (0.99–0.99)	<0.001		
Surfactant	2.86 (1.54–5.32)	0.001		
Days since rupture of membranes	1.01 (1.01–1.03)	0.010		
Day of life 3			0.891 (0.792–0.989)	0.863 (0.760–0.967)
Birth weight (grams)	0.99 (0.99–0.99)	<0.001		
Surfactant	2.74 (1.48–5.07)	0.001		
FiO_2_	1.03 (1.02–1.04)	<0.001		
Day of life 7			0.930 (0.867–0.993)	0.918 (0.849–0.988)
Birth weight (grams)	0.99 (0.99–0.99)	<0.001		
Surfactant	3.42 (1.89–6.19)	<0.001		
FiO_2_	1.00 (0.98–1.02)	0.830		
Day of life 14			0.956 (0.910–0.999)	0.949 (0.901–0.998)
Birth weight (grams)	0.99 (0.99–0.99)	<0.001		
Surfactant	3.55 (1.97 –6.41)	<0.001		
FiO_2_	1.01 (0.98–1.03)	0.610		

CI, confidence interval; AUC, time-dependent area under the receiving-operating-curve for *t* = 36 weeks; BCV, bootstrap cross-validation; FiO_2_, fraction of inspired oxygen; sHR, subhazard ratio.

**Figure 2:**
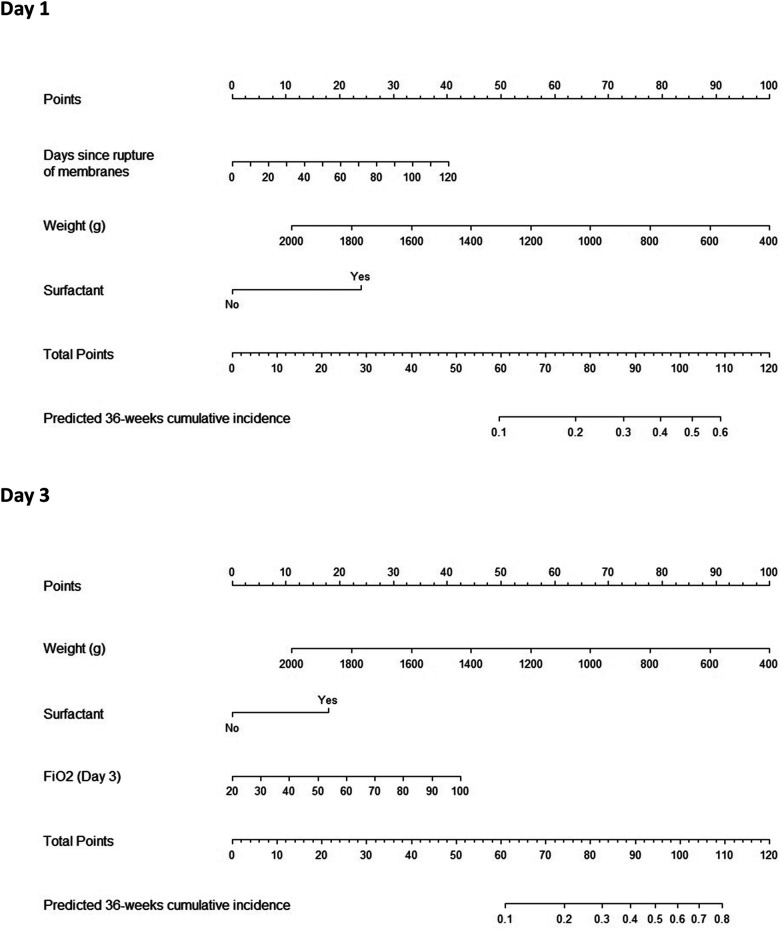
Nomograms predicting bronchopulmonary dysplasia (BPD) at postnatal days 1 (**A**) and 3 (**B**). Specific values for individual patients are located on each variable axis. A line is drawn upward to the “Points” axis to determine the number of points corresponding to each variable. The sum of points for all variables is calculated, and a line drawn downwards to the probability axis to determine the risk of BPD.

Figure 2 shows the nomograms developed based on the multivariate competing risk models for postnatal days 1 and 3. The AUC scores for the predictions at day of birth and DOL 3 were 0.896 (95% CI, 0.792–0.999) and 0.891 (95% CI, 0.792–0.989), respectively. Optimism adjusted AUC values for the same models were 0.881 (95% CI, 0.767–0.995) and 0.863 (95% CI, 0.760–0.967), showing good predictive accuracy (Supplementary Figures S2A,B), especially in babies 28–32 weeks GA, being the discriminative ability of the models lower for children <28 weeks GA (Supplementary Table S2).

For each of the nomograms, the estimated probability of BPD was obtained for all cases in the database, and newborns were categorized into four risk groups according to the quartiles of the estimated BPD probabilities. Differences between CIF of BPD curves by risk subgroups were statistically significant (Supplementary Figure S3). The calibration curves of both nomograms are also shown in Supplementary Figures 2C,D. Subgroup analysis (Supplementary Figure S3) showed that the observed percentages of BPD cases were consistent with the estimated probabilities.

The decision curves of the competing risk nomograms are also displayed in Supplementary Figures 2E,F.

## 4 Discussion

The competing risk analysis presented here shows that BPD can be effectively predicted using simple models that include clinical variables collected at the bedside during the first 3 days of life. To our knowledge, this is the first study to use this methodology to develop predictive models for BPD.

The importance of the availability of good prediction tools in neonatology has been previously highlighted, both for clinical practice and research (36). However, despite the efforts made by researchers and clinicians, BPD is still a major concern for health care providers and families, partly due to a lack of widely used prediction tools. Critical analysis of previous research on predictive models identified the exclusion of infants that die before BPD diagnosis and the use of combined outcomes as areas of potential improvement (10).

The explanation of this problem entails the introduction of two statistical concepts: time-to-event analysis and competing risks. In many studies, the results are assessed longitudinally, and each patient in the cohort is evaluated for a period of time until the event occurs. The study objectives can be to estimate the probability of occurrence of the event or its association with other variables of interest. An event that hinders or modifies the possibility of observing the event of interest is known as a competing event (37). In the case of BPD, patients who die prior to BPD diagnosis are no longer at risk and cannot be treated either as individuals with BPD or as healthy patients, and therefore death constitutes a competing risk for BPD.

Excluding patients who die, as was previously done (14), may not be correct. In fact, the most serious cases of lung disease of prematurity are probably found among those who died in the first days of life, and therefore may have been candidates for early treatment. For this reason, some studies include deaths from respiratory causes among patients with severe BPD (38). However, this also may be methodologically incorrect, as some patients are assigned a theoretical and somewhat arbitrary diagnosis. By contrast, other studies include infants who die in the non-BPD group (39), and consider them as members of the “healthy” controls.

Importantly, a recent systematic review and meta-analysis of BPD prediction models (10) discussed the importance of taking BPD and mortality into account as competing risks, although the authors propose using combined outcomes to address this limitation. In our opinion, the use of combined outcomes results in the attribution of common risk factors to two very different events. The literature includes studies of risk-factors for mortality in premature infants, the results of which differ from those focusing on BPD risk-factors (40, 41). This indicates that, although both variables share several common risk factors, there are many others that are unique to one or the other, since there are multiple causes of death that do not share the same pathophysiological pathways of BPD (40–42).

Competing risk analysis is a special type of survival analysis that aims to correctly estimate the marginal probability of an event in the presence of competing events. To the best of our knowledge, this methodology has not been previously used to predict BPD. A similar analysis focusing on another relevant complication of preterm birth, retinopathy of prematurity, was carried out by Miller et al. (43). This disease has similarities with BPD, since its development is determined by variables in the first days of life, before diagnosis is made weeks later (diagnosis cannot be established in patients who die earlier) (44). Moreover, in a recent clinical trial of hydrocortisone in BPD, the combined variable death or BPD at 36 weeks was used as the main study outcome, but an analysis using competitive risk models was also planned by the investigators, showing the relevance of this methodology in neonatal clinical trials (45).

The results of our models showed that predictive capacity was best established on DOL 1 using simple and readily accessible variables: birth weight, surfactant requirement, and time since rupture of membranes. On DOL 3, the variables ultimately included in the model were birth weight, surfactant requirement, and FiO_2_. The high discriminatory capacity of the proposed models, in terms of AUC values, could be surprising given the reduced number of variables included. It should be noted that the use of a competing risks methodology could provide a more unbiased estimate of the probability of BPD and the HR associated with each of the covariates considered, which may lead to a better predictive ability. On the other hand, all variables finally included in the models have been previously described in the literature as risk factors for the disease and are biologically plausible (9). In this respect, we would like to highlight the simplicity of our models, which consist of a limited number of objective clinical variables, recorded at bedside during the first days of life. Our data show that this simplicity does not imply inferior predictive capacity relative to more complex models that include more variables or biochemical or even ultrasound markers (46–49).

The aforementioned systematic review by Peng et al. provided some recommendations for the development of future models (9). The authors highlighted the importance of models using variables that are recorded sufficiently early, thereby enabling initiation of preventive treatments and/or recruitment of patients in clinical trials.

The present findings are clinically relevant, as the sooner high-risk patients are identified, the sooner preventive treatment can be instituted. Hallmark studies indicate that the benefits of treatment outweigh the risks in high-risk patients (50) when the estimated probability of experiencing BPD is high [>40% (95% CI 33%–46%)] (51). Our findings indicate that if a patient has received surfactant in the first hours of life, the individual risk of BPD can be effectively predicted based on birth weight and time since rupture of membranes or FiO_2_ on DOL 3 (Figure 2), without needing to wait for subsequent respiratory progression, when many treatments would be futile and heterogeneity in clinical practice may be greater.

Limitations of our study include the smaller sample size relative to other studies: this implies a limited number of events, making it difficult to explore the utility of more complex models, and may explain why we did not obtain better results on days 7 and 14. Moreover, our study was carried out using data from a retrospective cohort from a single center, without external validation. In particular, the sample studied is relatively mature with a mean gestational age of 29.3 weeks, so results could be not generalizable to more immature babies, as suggested by the AUC values obtained in the subset of babies <28 weeks GA, albeit with a very small sample size. This may also be related to the results of the calibration of the models, which show better performance in children at low risk of BPD and poorer calibration in babies at medium to high BPD risk, probably coinciding with those with a lower GA. External validation is currently underway using a national population-based cohort, and we hope to have data in the coming months. Finally, some limitations of the proposed methodology cannot be overlooked. A time-to-event analysis with PMA (which includes GA) as the time variable prevents us from analysing the effect of GA as an additional covariate in the proposed models. However, it can be adjusted for birth weight, which is a closely related variable. On the other hand, the proposed models include variables collected on specific days of life that may correspond to different PMA for different children, so it would be interesting to explore how to incorporate these time-dependent variables in a more efficient way using an analogous methodology.

We believe that the methodology proposed and the results produced using simple models based on early clinical data could be of great interest to the scientific community in general, and the field of clinical neonatology in particular. In conclusion, our results show that BPD can be effectively predicted using models based on variables that can be measured during the first hours of life, including birth weight, surfactant requirement, time since membrane rupture, and FiO_2_ in the first 3 days of life. Further research is needed to externally validate our findings and continue the quest to develop high-quality tools to predict BPD.

## Data Availability

The raw data supporting the conclusions of this article will be made available by the authors, without undue reservation.
