# Supplementary material for: Prediction of bronchopulmonary dysplasia in very preterm infants: competitive risk model nomogram
Source: Front Pediatr. 2024 Feb 20;12:1335891. doi: 10.3389/fped.2024.1335891 (PMC10912561; doi:10.3389/fped.2024.1335891)

**SUPPLEMENTARY MATERIALS**

**Supplementary Table 1.** Univariable competing-risk regression to estimate risk factors for death.

**Supplementary Table 2.** Discriminant ability of developed models according to gestational age.

**Supplementary Figure 1.** Estimation of the cumulative incidence probability for bronchopulmonary dysplasia (BPD) and death by using the competing risk method.

**Supplementary Figure 2.** Time-dependent ROC curve analysis (A, B), calibration plots (C,D) and decision curve analysis (E, F) for BPD prediction based on the nomograms for postnatal days 0 (first column) and 3 (second column).

**Supplementary Figure 3.** Estimation of the cumulative incidence probability for bronchopulmonary dysplasia (BPD) for the stratified risk groups according to (A) Nomogram for postnatal day 0. (B) Nomogram for postnatal day 3.

**Supplementary Table 1.** Univariable competing-risk regression to estimate risk factors for death.

|  | **Death-sHR (95% CI)** | **p-value** |
| --- | --- | --- |
| **DAY OF LIFE 1** |  |  |
| **Maternal age (years)** | 1.04(0.97-1.11) | 0.190 |
| **Maternal arterial hypertension** | 1.31(0.58-2.96) | 0.500 |
| **Maternal smoking** | 0.52(0.12-2.20) | 0.380 |
| **Multiple birth** | 0.43(0.17-1.07) | 0.072 |
| **IFV** | 1.03(0.42-2.55) | 0.940 |
| **Female** | 0.75(0.36-1.56) | 0.450 |
| **Chorioamnionitis** | 2.42(1.10-5.34) | 0.028 |
| **Prenatal steroids** | 0.22(0.08-0.56) | 0.002 |
| **Caesarean section** | 1.14(0.48-2.68) | 0.760 |
| **Days since rupture of membrane** | 0.98(0.93-1.02) | 0.360 |
| **Birth weight (grams)** | 0.99(0.99-0.99) | 0.000 |
| **Birth weight Z-score** | 1.00(0.98-1.02) | 0.460 |
| **Apgar 1 minute** | 0.67(0.57-0.79) | 0.000 |
| **Apgar 5 minute** | 0.70(0.60-0.83) | 0.000 |
| **Oxygen in DR** | 2.36(0.55-10.0) | 0.250 |
| **Intubation in DR** | 2.35(1.11-4.95) | 0.024 |
| **Chest compressions in DR** | 4.73(1.89-11.8) | 0.001 |
| **Adrenaline in DR** | 3.52(1.21-10.2) | 0.020 |
| **Temperature on admission (ºC)** | 0.95(0.90-1.01) | 0.120 |
| **Surfactant** | 13.5(3.22-56.8) | 0.000 |
| **Age at surfactant administration (hours)** | 0.91(0.82-1.02) | 0.130 |
| **DAY OF LIFE 3** | | |
| **FiO_2_** | 1.03(1.00-1.05) | 0.012 |
| **MV** | 25.9(8.62-78.0) | 0.000 |
| **MV during the first 72 hours of life** | 14.5(5.91-36.0) | 0.000 |
| **PDA** | 0.94(0.13-6.76) | 0.950 |
| **Nosocomial infection** | - | - |
| **DAY OF LIFE 7** | | |
| **FiO_2_** | 1.13(1.08-1.18) | 0.000 |
| **MV** | 34.4(9.23-128.) | 0.000 |
| **MV during the first week** | 15.3(5.35-44.1) | 0.000 |
| **PDA** | 1.44(0.50-4.11) | 0.500 |
| **Nosocomial infection** | 1.61(0.49-5.33) | 0.430 |
| **DAY OF LIFE 14** | | |
| **FiO_2_** | 1.06(1.04-1.07) | 0.000 |
| **MV** | 24.1(4.54-128.) | 0.000 |
| **MV during the first two weeks** | 9.41(2.27-38.9) | 0.002 |
| **MV during 3-14 day of life** | 11.0(3.28-37.1) | 0.000 |
| **PDA** | 1.22(0.46-3.19) | 0.680 |
| **Nosocomial infection** | 1.63(0.74-3.55) | 0.220 |
| Death-sHR= death-related subhazard ratio; IVF= in vitro fertilization; IGR= intrauterine groth restardation; DR= delivery room; MV= Mechanical ventilation; NIV= Non-invasive mechanical ventilation; PDA= patent ductus arteriosus. | | |

**Supplementary Table 2.** Discriminant ability of developed models according to gestational age.

|  | **<28 weeks GA** | | **28-32 weeks GA** | |
| --- | --- | --- | --- | --- |
|  | **AUC (95% CI)** | | **AUC (95% CI)** | |
|  | **Apparent** | **BCV-corrected** | **Apparent** | **BCV-corrected** |
| **Nomogram for postnatal day 1** | 0,633  (0,503-0,763) | 0,399  (0,266-0,533) | 0,945  (0,863-1,00) | 0,863  (0,709-1,00) |
|  |  |  |  |  |
| **Nomogram for postnatal day 3** | 0,665  (0,539-0,790) | 0,552  (0,421-0,682) | 0,907  (0,771-1,00) | 0,846  (0,667-1,00) |
|  |  |  |  |  |
| **Nomogram for postnatal day 7** | 0,718  (0,596-0,840) | 0,632  (0,500-0,763) | 0,973  (0,922-1,00) | 0,990  (0,971-1,00) |
|  |  |  |  |  |
| **Nomogram for postnatal day 14** | 0,747  (0,625-0,868) | 0,714  (0,585-0,843) | 0,977  (0,927-1,00) | 0,911  (0,800-1,00) |

**Supplementary Figure 1.** Estimation of the cumulative incidence probability for bronchopulmonary dysplasia (BPD) and death by using the competing risk method.

**
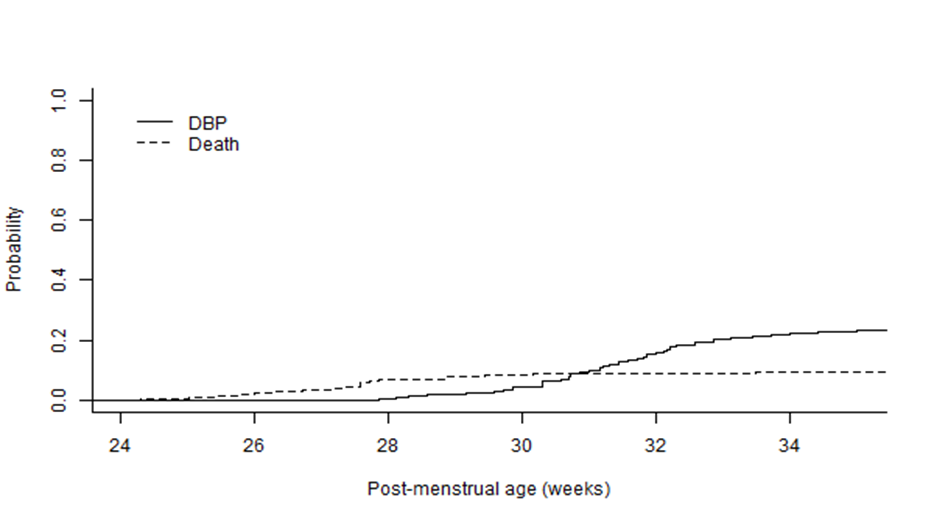
**

**Supplementary Figure 2.** Time-dependent ROC curve analysis (A, B), calibration plots (C,D) and decision curve analysis (E, F) for BPD prediction based on the nomograms for postnatal days 1 (first column) and 3 (second column).

|  | **Day of life 1** |  | **Day of life 3** |
| --- | --- | --- | --- |
| **A** | 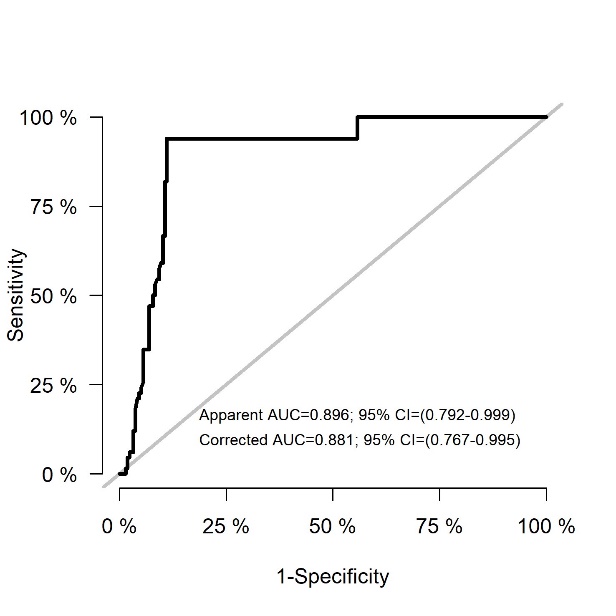 | **B** | 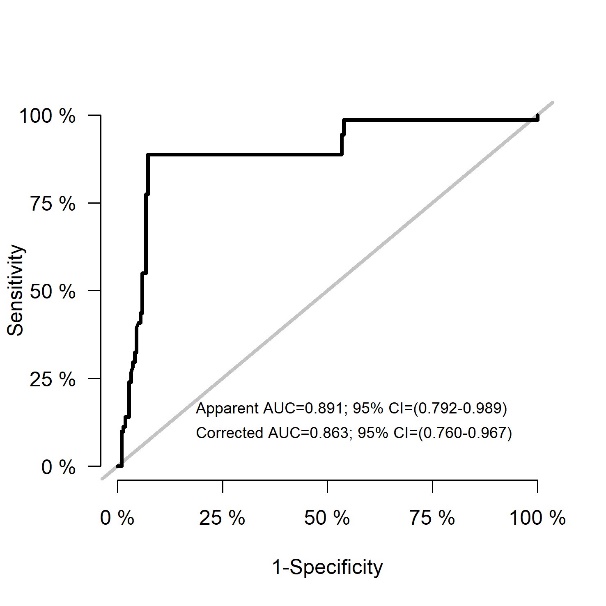 |
| **C** | 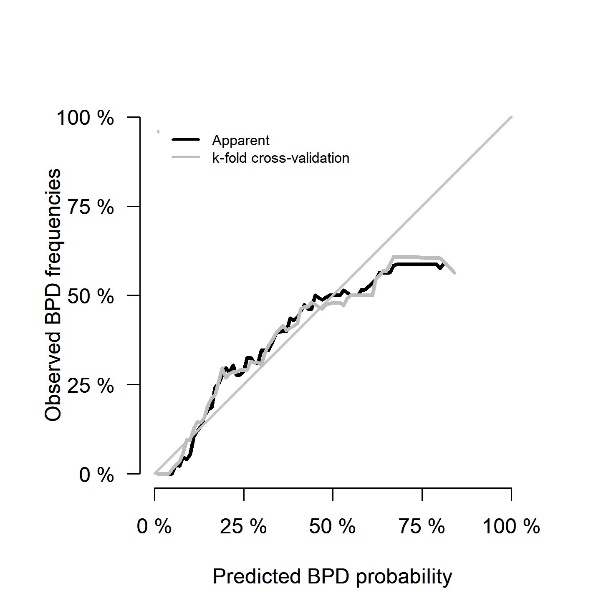 | **D** | 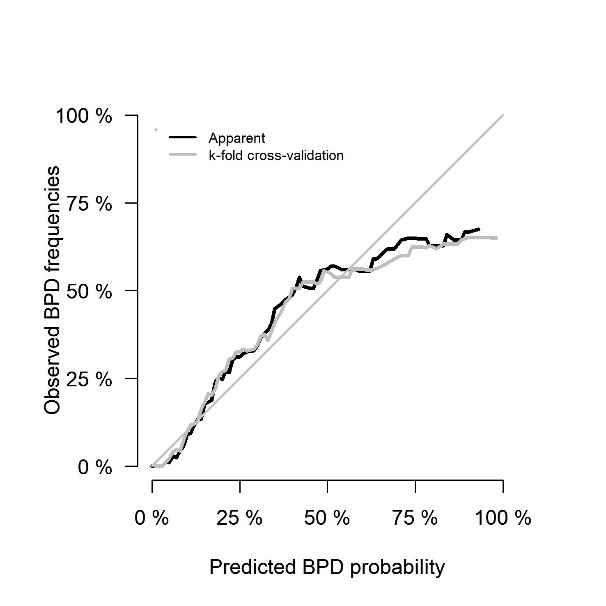 |
| **E** | 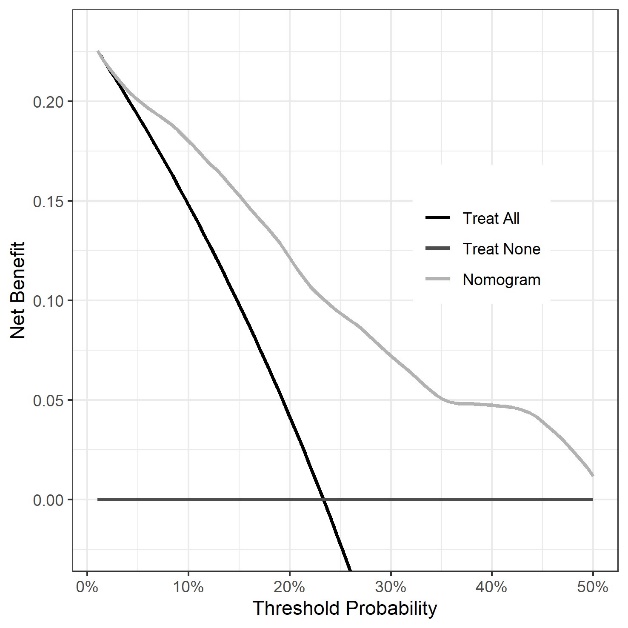 | **F** | 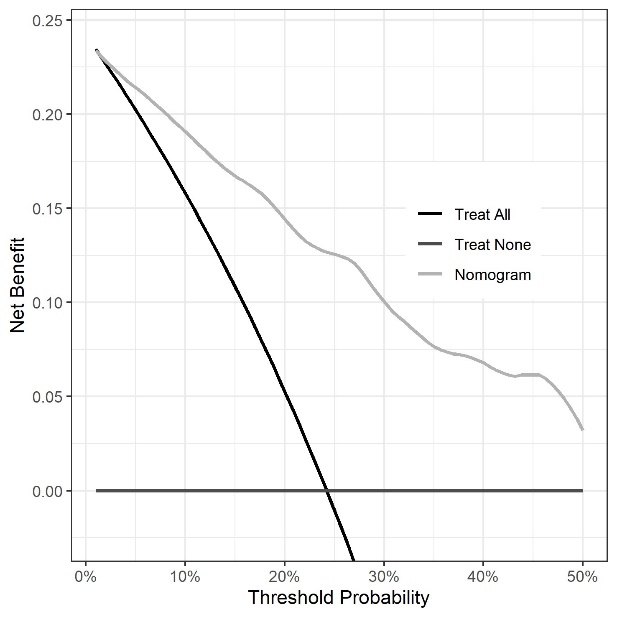 |

**Supplementary Figure 3.** Estimation of the cumulative incidence probability for bronchopulmonary dysplasia (BPD) for the stratified risk groups according to (A) Nomogram for postnatal day 1. (B) Nomogram for postnatal day 3.

**A**


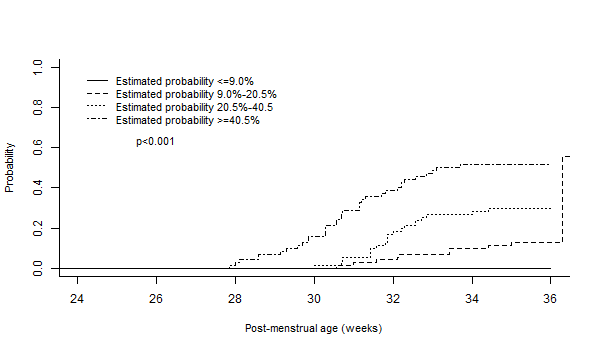


**B**


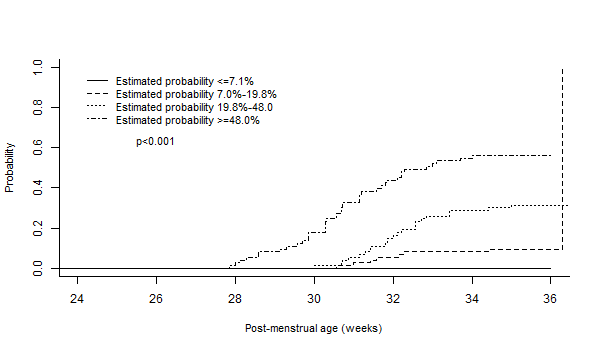

Supplement: Supplementary file 1 [file Table1.docx]
